# Whole Exome Sequencing Study Identifies Distinct Characteristics of Transformed Small Cell Lung Cancer With EGFR Mutation Compared to De Novo Small Cell and Primary Non‐Small Cell Lung Cancers

**DOI:** 10.1002/cam4.70838

**Published:** 2025-04-08

**Authors:** Jinjing Tan, Dan Zhao, Qunhui Wang, Yanjing Peng, Jie Li, Xi Li, Nanying Che, Ying Hu, Hua Zheng

**Affiliations:** ^1^ Cancer Research Center, Beijing Chest Hospital Capital Medical University, Beijing Tuberculosis and Thoracic Tumor Research Institute Beijing China; ^2^ Department of Pathology, Beijing Chest Hospital Capital Medical University, Beijing Tuberculosis and Thoracic Tumor Research Institute Beijing China; ^3^ Department of Medical Oncology, Beijing Chest Hospital Capital Medical University, Beijing Tuberculosis and Thoracic Tumor Research Institute Beijing China

**Keywords:** de novo small cell lung cancer, EGFR mutation, EGFR‐TKIs targeted therapy, genomic alterations, lung adenocarcinoma, transformed small cell lung cancer, whole exome sequencing

## Abstract

**Background:**

Epidermal growth factor receptor (EGFR)‐mutated lung adenocarcinoma (LUAD) is the most common subtype among non‐small cell lung cancer (NSCLC) and targeted therapies are the primary approach for treatment. However, the development of resistance to therapy and histological transformation into small cell lung cancer (SCLC) present significant challenges. Understanding the mechanisms underlying this transformation is crucial for effective differential diagnosis and the formulation of treatment strategies.

**Methods:**

In this study, we collected tissue from 5 primary LUAD before SCLC transformation, 12 transformed SCLC after EGFR tyrosine kinase inhibitor (TKI) treatment, and 18 de novo SCLC from lung cancer patients treated at Beijing Chest Hospital, Capital Medical University from January 2015 to December 2021. Whole‐exome sequencing was performed on these samples to compare the genomic alterations of these three tumor types, elucidating their similarities, differences, and connections. Statistical analyses were conducted using the Fisher exact test and performed with R v4.2.1 environment.

**Results:**

Among 12 transformed SCLC cases, the majority were female (10/12, 83.3%), non‐smokers (10/12, 83.3%) and harbored EGFR 19del mutations (11/12, 91.7%). Four were with limited stage and 8 with extensive stage. TP53 mutations and RB1 loss are important but not necessary for SCLC transformation. The mutation rates of TP53 were 60% (3/5) in primary LUAD, 70% (7/10) in transformed SCLC, and 89% (16/18) in de novo SCLC. RB1 loss rates were 40% (2/5) in primary LUAD, 30% (3/10) in transformed SCLC, and 50% (9/18) in de novo SCLC. Additionally, mutations in COL22A1 and ALMS1 were only observed in transformed SCLC and de novo SCLC. In contrast, mutations in PTCH2, CNGB3, SPTBN5, CROCC, and MYO15A were more common in transformed SCLC, whereas PABPC3 and MUC19 mutations were more frequent in de novo SCLC. Smoking‐related mutations (SBS4) were only found in de novo SCLC, with no changes observed in transformed SCLC. TMB levels were significantly lower in transformed SCLC compared to de novo SCLC (*p* = 0.01). Genomic instability was significantly higher in transformed SCLC compared to primary LUAD and de novo SCLC. This was supported by higher levels of homologous recombination deficiency (HRD, *p* = 0.025), uniparental disomy (UPD, *p* = 0.003), loss of heterozygosity (LOH, *p* = 0.008), and telomeric allelic imbalance (TAI, *p* = 0.02). The increased frequency of UPD events in transformed SCLC suggests that UPD may act as a “second hit” in Knudson's model, leading to biallelic inactivation of tumor suppressor genes. High similarity was observed in genetic alterations related to DNA damage repair (DDR) and Notch signaling pathways between transformed SCLC and de novo SCLC.

**Conclusions:**

The identification of these specific genomic alterations in transformed SCLC contributes to a better understanding of the mechanisms driving this transformation. This knowledge may guide future predicting the transformation of SCLC and the development of personalized treatment strategies for these patients.

## 1 Introduction

Lung cancer is the most commonly diagnosed cancer type globally and leads the first mortality among all cancer types worldwide [1]. Based on the histological characteristics of the tumor, lung cancer can be divided into two main subtypes: small cell lung cancer (SCLC; approx. 15%) and non‐small cell lung cancer (NSCLC; approx. 85%). NSCLC is further divisible into two main histological subtypes: lung adenocarcinoma (LUAD) and lung squamous cell carcinoma (LUSC), along with several less frequently observed subtypes, such as large cell carcinoma and, adeno‐squamous. Lung cancer histological subtype classification mainly depends on the pathological morphology and immunohistochemistry of tumor tissue, reflecting the differences in tumor origin and biological behavior [2]. It is widely believed that LUAD develops from alveolar type II (AT2) epithelial cells or cells within bronchioalveolar duct junctions and could be identified using marker proteins TTF‐1 and CK7, which are indicative of LUAD to aid diagnosis [3]. Yet SCLC may arise from mature cells in the neuroendocrine lineage. Histological transformation to SCLC from NSCLC when resistance to EGFR Tyrosine Kinase Inhibitors (EGFR‐TKIs) has been reported in 3%–15% of cases in repeated biopsy series [4, 5, 6]. The exact mechanisms leading to this pathological transformation are not well understood, but two main hypotheses have been proposed [5]. Some scholars suppose that patients may initially have mixed small cell carcinoma, but due to limitations in the biopsy samples during diagnosis, the small cell component cannot be detected [5, 6]. Through drug screening, SCLC becomes the predominant clone. The other hypothesis suggests that transformed SCLC is derived from a lineage evolution of adenocarcinoma cells. An epigenetic study found that the miRNA expression profile of transformed SCLC retains characteristics of adenocarcinoma while also possessing features of de novo small cell carcinoma [7, 8], which supports this hypothesis. Since SCLC transformation detected sporadically, there are no clear clinical guidelines for the treatment of transformed SCLC [9]. Exploring the origin of transformed SCLC and its development is not only of academic significance on tumor evolution but also of great clinical value in determining the treatment strategy. At present, in vitro modeling of LUAD to SCLC transformation has been established to study how the transformation occurs [10].

In order to address the limitations of existing research, we collected primary LUAD tissue with EGFR mutation before SCLC transformation, transformed SCLC tissue after EGFR‐TKI treatment, and de novo SCLC tissue, and performed whole‐exome sequencing on these samples. Our aim is to compare and elucidate the similarities, differences, and connections among these three pathological tumor types from the perspective of genomic alterations, with the hope of providing insights for future diagnosis and treatment of transformed SCLCs.

## 2 Methods

### 2.1 Clinical Cohort

The study samples were collected from the patients with NSCLC who received antitumor therapy at Beijing Chest Hospital, Capital Medical University from January 2015 to December 2021. For the primary LUAD and transformed SCLC cohorts, the inclusion criteria were as follows: (1) initial histological or cytological diagnosis of LUAD; (2) EGFR mutation was confirmed by Amps qPCR or NGS testing of the biopsy tissue or blood; (3) have received prior EGFR‐TKIs targeted therapy; (4) after drug resistance, a second biopsy showing SCLC confirmed by pathological test; (5) complete clinical information such as medical history, imaging examination, TNM staging and therapeutic process and efficacy evaluation; and (6) paraffin‐embedded sections with biopsy tissue or embedded pleural effusion containing more than 90% tumor, with sufficient tissue quantity for library construction and genome sequencing. A total of 5 primary LUAD tissues before SCLC transformation and 12 transformed SCLC tissues after resistance to EGFR‐TKIs treatment were obtained for subsequent whole exome sequencing analysis. For the de novo SCLC cohort, the inclusion criteria were as follows: (1) initial histological or cytological diagnosis of SCLC; (2) no prior treatment had been performed; (3) complete clinical information such as medical history, imaging examination and stage; and (4) paraffin‐embedded sections with surgical tissue containing more than 90% tumor and sufficient tissue volume for library construction and genome sequencing. We randomly selected 18 patients with de novo SCLC for subsequent whole exome sequencing analysis. The clinical features of all patients are detailed in Table S1. This retrospective study was approved and informed written consent was waived by the Ethics Committee at Beijing Chest Hospital, Capital Medical University. The research was conducted in compliance with the Declaration of Helsinki.

### 2.2 Sample Collection and DNA Extraction

All tumor samples were obtained from paraffin‐embedded sections of surgical, biopsy or embedded pleural effusion specimens. Hematoxylin and eosin (H&E) staining, along with histological microscopy, were conducted on these sections. Expert pathologists identified the tissue type and assessed the tumor purity. Genomic DNA was extracted from formalin‐fixed paraffin‐embedded (FFPE) tumor samples using the QIAamp DNA FFPE Tissue Kit (Qiagen GmbH, Hilden, Germany), following the manufacturer's instructions. The DNA concentration was determined using the Qubit dsDNA HS (High Sensitivity) assay kit with a Qubit fluorometer (Invitrogen; Thermo Fisher Scientific Inc., Waltham, MA, USA). To evaluate DNA integrity, 200 ng of the extracted DNA was loaded onto a 1% agarose gel alongside a DNA marker (l‐Hind III) (Takara Biotechnology Co. Ltd., Dalian, China). Samples were deemed intact if they exceeded the second‐largest bands (9416 bp) of the l‐Hind III marker and were then used for further analysis. All genomic DNA was extracted from the tissue samples, and quality control was performed before sequencing. Two cases of transformed SCLC tissue did not pass quality control and were excluded from sequencing, as noted in Table S2.

### 2.3 Whole Exome Sequencing

For each sample, 1.0 μg of genomic DNA was used as the starting material for library preparation. Sequencing libraries were constructed using the Agilent SureSelect Human All Exon V6 kit (Agilent Technologies, San Diego, CA) according to the manufacturer's guidelines, and index codes were assigned to each sample. Briefly, DNA fragmentation was performed using a hydrodynamic shearing system (Covaris, Massachusetts, USA) to produce fragments ranging from 180 to 280 bp. The resulting overhangs were converted to blunt ends through exonuclease and polymerase activities, after which the enzymes were removed. Following the adenylation of the 3′ ends of the fragments, adapter oligonucleotides were ligated. Fragments with adapters on both ends were then selectively amplified via PCR. After amplification, the library was hybridized in a liquid phase with biotin‐labeled probes, and streptavidin‐coated magnetic beads were utilized to capture the exons of the target genes. The captured libraries underwent additional PCR enrichment to add index tags for hybridization preparation. Final products were purified using the AMPure XP system (Beckman Coulter, Beverly, USA) and quantified using the Agilent high sensitivity DNA assay on the Agilent Bioanalyzer 2100 system. The qualified exome capture libraries were then sequenced on the Illumina NovaSeq 6000 platform, following standard protocols for 150 bp paired‐end multiplexed sequencing.

### 2.4 Processing of WES Sequencing Data

After filtering out low‐quality sequencing reads and adapter sequences using FASTP, clean reads were aligned to the human reference genome (UCSC hg19) with the Burrows‐Wheeler Aligner (bwa‐0.7.17). The aligned genomes were sorted using Sambamba (v0.6.7), and duplicate reads were identified with Picard tools (v2.18.9). Somatic single nucleotide variants (SNVs) and insertions/deletions (INDELs) were identified using VarScan2 and MuTect2 in tandem. Specifically, VarScan2 somatic (v2.3) was employed for variant calling with standard parameters, except for adjustments: minimum coverage was set to 10 for normal samples and 8 for tumor samples, the minimum variant frequency was set to 0.01, and tumor purity was established at 0.5. For the MuTect2 analysis, we utilized the version included in the GATK bundle (4.0.5.1) with default settings. Functional annotation of the variants was performed using ANNOVAR tools.

### 2.5 SNV and INDEL Filtering

To minimize false positive variant calls, additional filtering methods were applied to the mutation detection results from both MuTect2 and VarScan2. A variant was considered valid if it was identified by both tools, with a somatic *p* value ≤ 0.1 for SNVs and ≤ 0.05 for INDELs, and a variant allele frequency (VAF) > 2%. Alternatively, a variant detected solely by VarScan2 needed a VAF exceeding 5%. For matched tumor sequencing data, the VAF should be less than 1%, and the number of reads for alternative alleles must be fewer than 5 for SNVs and 2 for INDELs. Additionally, variants found in regions of simple repeats and segmental duplications were excluded. The population frequency of the SNV was required to be below 1% in any of the following databases: 1000 Genomes, EXAC, or ESP6500. For tumors without paired blood samples, a more stringent criteria was used to remove any possible germline mutations detected on SCLC or NSCLC driver genes. First, a panel of 17 normals was used to build a PON to filter somatic variant calls from artifacts and germline leakage. Then, variants with population allelic frequency greater than 0.001 in 1000 genomes or gnomAD databases were removed. Moreover, only pathogenic coding mutations, which were evaluated using SIFT or Polyphen as damaged, and truncated mutations were reserved for further analysis.

### 2.6 CNV Analysis

We employed ASCAT to assess somatic copy number alterations (SCNA) in paired tumor‐normal sequencing data. The tumor purity and ploidy estimates were further refined through manual validation of the ABSOLUTE results. Allele counts at specific positions were derived from the 1000 Genomes Project using AlleleCounter, with a minimum coverage of 20 for the normal samples applied during filtering. LogR (the depth ratio of tumor to normal) values underwent GC wave correction via ASCAT, retaining only heterozygous B‐allele frequency (BAF) values for subsequent analysis. Allele‐specific segmentation was conducted to produce segmented logR and BAF data using ascat.aspcf. Manual checks were performed to determine the optimal model for ploidy and cellularity based on ABSOLUTE findings. For tumors sequenced without matched normal samples, CNVkit was utilized to identify copy number variations (CNV) with default parameters, excluding a pooled reference created from multiple FFPE normal samples. Allele‐specific copy number was inferred from GATK variant calling results with threshold parameters set at −1.1, −0.4, 0.3, and 0.7. Significant somatic CNVs were identified using GISTIC2.0 based on outputs from ASCAT or CNVkit, with significant copy number alteration (CNA) regions defined as having amplifications or deletions with a residual *q* value of < 0.05.

### 2.7 HRD Score Calculation

Homologous recombination deficiency (HRD) scores are calculated using the scarHRD R package. The HRD score, derived from allele‐specific copy number data, combines the scores for loss of heterozygosity (LOH), telomeric allelic imbalance (TAI), and large‐scale transitions (LST). The HRD‐LOH score reflects the count of 15 Mb regions exhibiting LOH that do not encompass entire chromosomes. The HRD‐TAI score indicates allelic imbalances that reach the telomeric ends of chromosomes. Lastly, HRD‐LST is characterized by chromosomal breaks occurring between adjacent regions of at least 10 Mb, with a maximum distance of 3 Mb separating them.

### 2.8 Analysis of Mutational Signatures

The mutational signatures were assessed using the R package deconstructSigs (v1.8.0). A multiple linear regression model was employed to evaluate the activity levels of predefined signatures. The contribution of each mutational signature for individual patients was compared against the COSMIC SBS signature V2 (https://cancer.sanger.ac.uk/cosmic/signatures_v2.tt).

### 2.9 Statistical Analysis

To compare continuous values across different groups, the Fisher exact test was utilized. Figures were generated using either the ggplot2 or ComplexHeatmap packages. All statistical analyses were conducted in the R environment (v4.2.1), with statistical significance set at a two‐sided *p* value of < 0.05.

## 3 Result

### 3.1 Clinical Characteristics of Patients

According to the process of SCLC transformation, we collected 5 adenocarcinoma tissue samples from EGFR‐mutated LUAD patients before SCLC transformation, 12 transformed SCLC tissue samples after EGFR‐TKI treatment, and 18 de novo SCLC tissue samples for whole exome sequencing analysis. All tested DNA samples were sourced from FFPE tissue sections from patient's surgical, biopsy tissues or embedded pleural effusion. The clinical characteristics are presented in Table S1. It can be observed that transformed SCLCs are predominantly found in females (2/12, 83.3%) and non‐smokers (2/12, 83.3%), whereas de novo SCLCs are predominantly found in males and smokers. This is consistent with the epidemiological characteristics of LUADs and SCLCs. Among the transformed SCLC cases, four cases were at the limited stage and eight cases were at the extensive stage. After an average treatment duration of 17.8 months, SCLC components were detected during patient biopsies when resistance to EGFR‐TKIs (Table S2). The pathological diagnosis were made by professional pathologists. As shown in Figure S1, transformed SCLC tissue shares the same morphology with de novo SCLCs, the tumor cells are small, round oval or spindle‐shaped with scant cytoplasm and ill‐defined cytoplasmic borders. Immunohistochemical staining for the typical SCLC marker CD56, Syn, Napsin A were positive. Among them, six cases were identified as pure SCLC, whereas the other six cases were identified as mixed type, containing both adenocarcinoma and SCLC components.

Among the 12 patients who underwent SCLC transformation, the EGFR mutation type was predominantly exon 19 deletion (11/12, 91.7%), with one cases exhibiting simultaneous T790M mutations and only one case of L858R mutation at their adenocarcinoma stage. Unfortunately, for the transformed SCLCs tissues, we could not obtain the paired primary LUAD tissues due to the limited amount or substandard quality.

### 3.2 Mutational Landscape of Transformed SCLCs With Primary LUAD and De Novo SCLC

Genomic DNA was extracted from all tissue samples, and quality control was performed prior to sequencing. Two cases of transformed SCLC tissue failed quality control and were not sequenced, as shown in Table S2. The average sequencing coverage is 99.9%, with an average sequencing depth of 158.89× and an average clean base yield of 30G.

**FIGURE 1:**
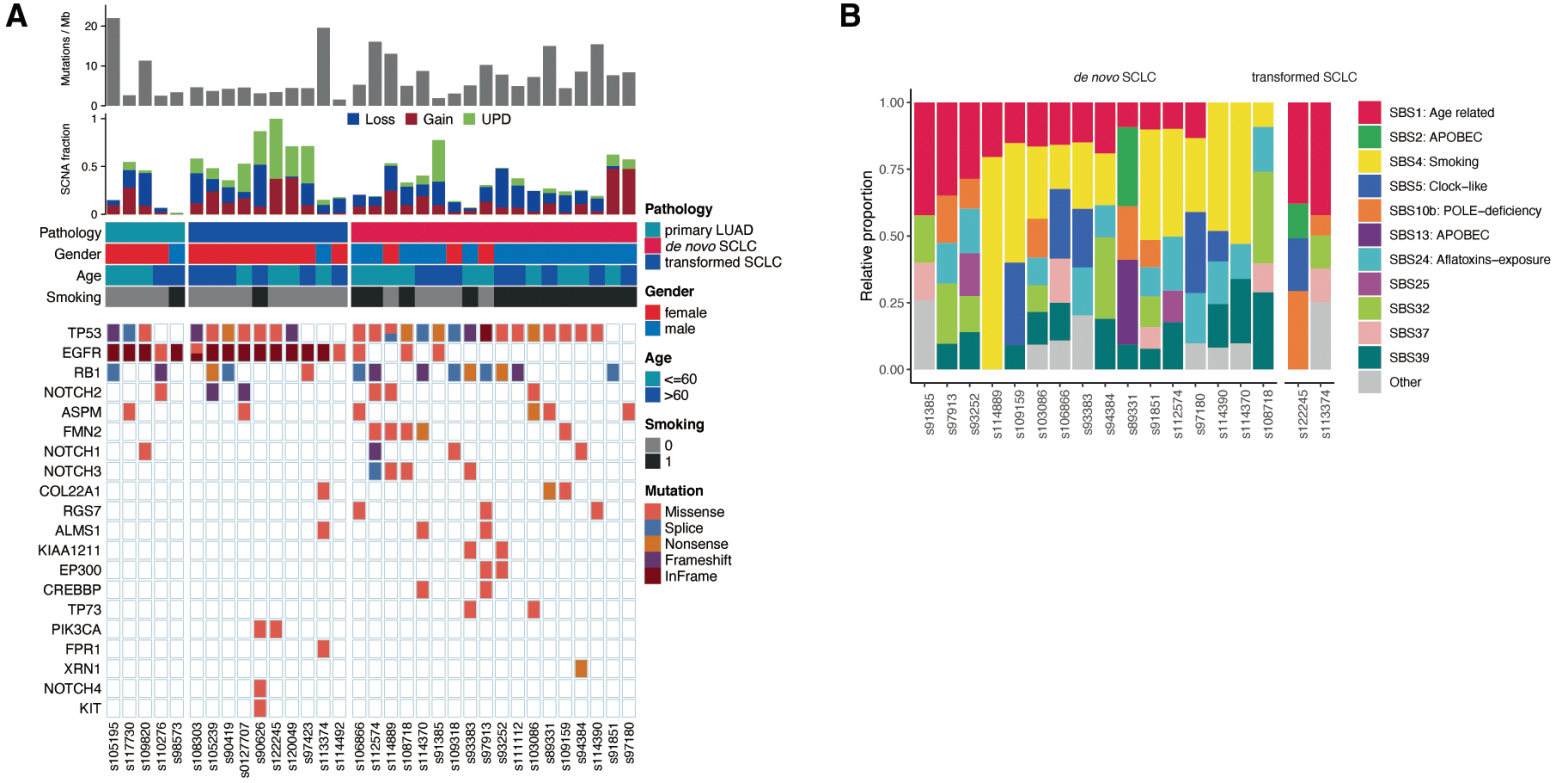
Somatic mutation landscape and somatic signature. (A) Heat map showing somatic mutation landscapes of three patient cohorts, that is, primary LUADs, transformed SCLCs and de novo SCLCs. Rows representing genes, while columns representing samples. Various kinds of somatic mutations were labeling with different colors as showing in legend. Along with the heat map, four clinical information were annotated in color tracks, including the pathology of samples, gender, age and smoking history of patients. In addition to that, TMB and SCNA fraction (CNV gain, CNV loss and UPD) were also displayed on the top of heat map. (B) Bar plot showing the components of somatic mutational signatures for each sample with color schema revealing the category. CNV, copy number variation; SCNA, somatic copy number alteration; SBS, single base substitution; TMB, tumor mutational burden; UPD, uniparental disomy.

Focusing on the somatic mutational landscape of primary LUADs, transformed SCLCs and de novo SCLCs, we found the mutational pattern of transformed SCLCs was more closely aligned with that of primary LUADs than with de novo SCLCs, especially for *EGFR* mutations (Figure 1A). In both primary LUADs and transformed SCLCs, all patients harbored at least one mutation in *EGFR*, whereas in de novo SCLCs, only 3 out of 18 (17%) patients harbored *EGFR* mutation. In the evolutionary process, transformed SCLCs reserved *EGFR* mutation originating from their own primary LUADs, which is a key distinguishing feature compared to de novo SCLCs. Additionally, the prevalence of *TP53* mutations showing a trend of increase among these three cohorts (Figure 1A), with 60%, 70% and 89% in primary LUADs, transformed SCLCs and de novo SCLCs, respectively. For another tumor suppressor gene, *RB1*, the prevalence didn't display obvious differences (Figure 1A), with 40%, 30%, and 50% of primary LUADs, transformed SCLCs and de novo SCLCs. Intriguingly, mutations in the genes *COL22A1* and *ALMS1* occurred only in transformed SCLCs and de novo SCLCs (Figure 1A), indicating their common roles in the originating of these two types of SCLCs.

To further confirm this observation, we also compared mutational landscape between our transformed SCLC cohort and three other published de novo SCLC cohorts (Figure S2). The similar phenomenon was observed, in which the prevalence of *TP53* mutations was comparable across all cohorts, whereas the prevalence of *EGFR* mutations was significantly higher in transformed SCLCs.

Next, we compared the components of somatic signatures between transformed SCLCs and de novo SCLCs. The age‐related signature (SBS1) appeared in almost all samples, regardless of whether they were transformed SCLCs or de novo SCLCs. On the other hand, the somatic signature associated with smoking (SBS4) was exclusively detected in de novo SCLCs (Figure 1B).

### 3.3 Distinct Pattern of Omics Features Between De Novo and Transformed SCLCs

**FIGURE 2:**
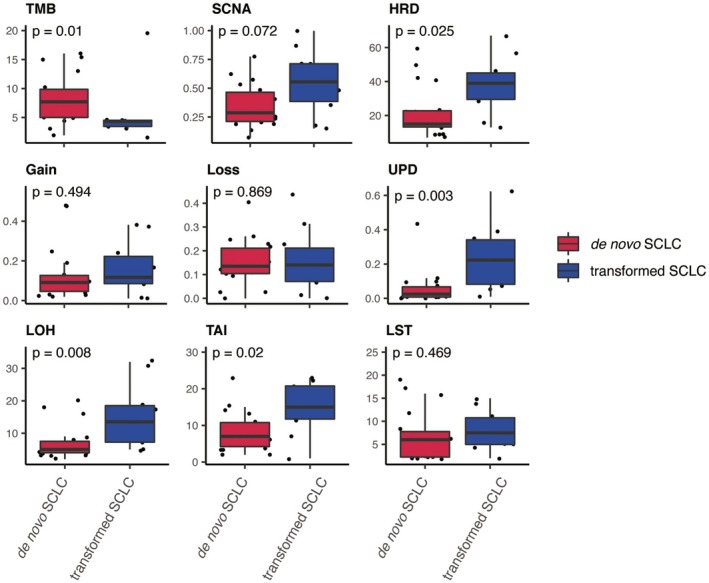
Comparison of omics features between de novo and transformed SCLCs. Nine panels showing the comparison of value distribution related to different omics features, including TMB, SCNA, HRD, CNV gain, CNV loss, UPD, LOH, TAI, and LST. The comparison was performed via Wilcoxon rank sum test and resulted *p* value was labeled on the top of each panel. Red box and blue box representing de novo and transformed SCLCs, respectively. CNV, copy number variation; HRD, homologous recombination deficiency; LOH, loss of heterozygosity; LST, large‐scale state transition; SCNA, somatic copy number alteration; TAI, telomeric‐allelic imbalance; TMB, tumor mutational burden; UPD, uniparental disomy. The HRD score is calculated by integrating LOH, LST, and TAI scores.

Based on a systematic comparison of omics features between de novo and transformed SCLCs, we identified the distinct pattern (Figure 2). First, the tumor mutational burden (TMB) was significantly higher in de novo SCLCs compared to that in transformed ones (Wilcoxon rank sum test, *p* value = 0.01). Second, in contrast, multiple omics features related to genomic instability were significantly higher in transformed SCLCs, including HRD (Wilcoxon rank sum test, *p* value = 0.025), uniparental disomy (UPD, Wilcoxon rank sum test, *p* value = 0.003), LOH (Wilcoxon rank sum test, *p* value = 0.008) and TAI (Wilcoxon rank sum test, *p* value = 0.02). The HRD score is the combination of loss of heterozygosity (LOH), telomeric‐allelic imbalance (TAI) and large‐scale state transitions (LST).

### 3.4 Extensive UPD Events Happened in Transformed SCLCs

**FIGURE 3:**
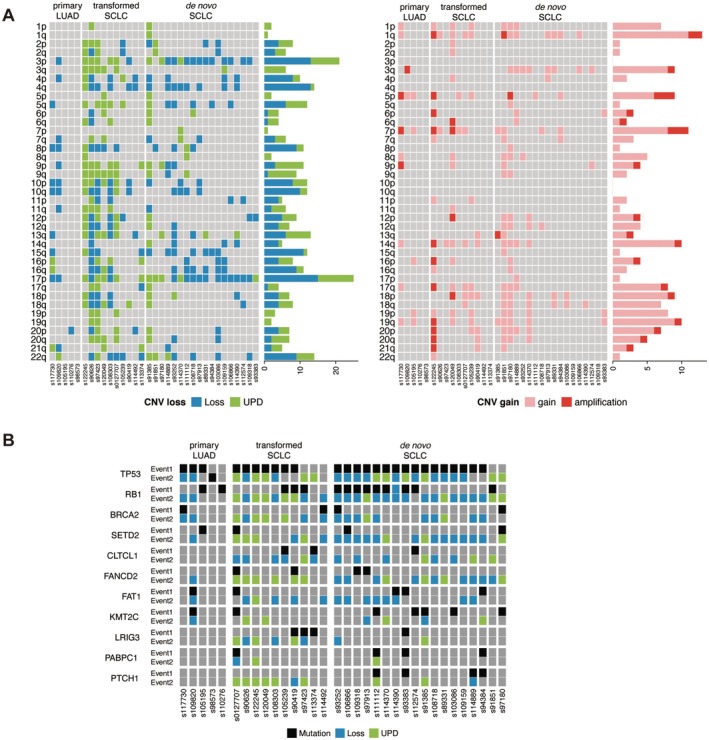
Somatic copy number alteration landscape and double‐hit events. (A) Two heat maps showing somatic copy number alteration landscape of CNV loss (left panel) and gain (right panel), respectively. Rows representing arm regions of 22 autosomal chromosomes and columns representing samples. Cohort of origin of samples were labeled on the top of heat map. Bar plot in the right of each panel showing the total number of CNV events for each arm region happened in all patients. (B) Heat map showing distribution of double‐hit events in cohorts of primary LUADs, transformed SCLCs and de novo SCLCs. Rows representing two genomic alterative events of genes and columns representing samples collected from different cohorts. Color schema showing the category of genomic alterative events. CNV, copy number variation.

As shown in the analysis of omics features, the UPD value was significantly higher in transformed SCLCs compared to de novo ones (Figure 2). Since UPD acts as a “second hit” in Knudson's model to achieve biallelic inactivation of tumor suppressor genes [11], we comprehensively compared CNV landscape between transformed SCLCs and de novo SCLCs to study this phenomenon in detail. Firstly, we analyzed the CNV events on chromosome arms. When focusing on chromosome arms with deleted CNV, we found that transformed SCLCs harbored significantly more UPD events compared to either primary LUAD or de novo SCLCs (Figure 3A). The extensive UPD events are likely an important driving factor for the oncogenesis of transformed SCLCs. Conversely, the distribution pattern of amplified CNV was similar among these three cohorts (Figure 3A). Meanwhile, the occurrence frequency of CNV gain events was lower than that of CNV loss events.

Since UPD has the potential to lead to homozygosity of existing aberrations such as mutations, deletions, methylations, histone‐modifications, or imprinted genes, it could contribute to the development and/or progression of cancer by inactivating tumor suppressor genes or doubling the copy number of oncogenic alleles [12]. Therefore, we integrated the mutational status with copy number loss and UPD profiles for each gene and selected the most frequently mutated ones to compare across the study cohort (Figure 3B). According to the hypothesis, the mutation was considered the first event (Event 1) and copy number loss and UPD were considered the second event (Event 2). Our results indicated that TP53 was simultaneously mutated and affected by UPD in both transformed SCLC and de novo SCLC. For transformed SCLCs, 50.0% of the samples showed a mutation at TP53 also presented copy number loss or UPD affecting 17p13.1, while for de novo SCLCs, the rate was 77.8%. As for mutation at RB1, both transformed SCLCs and de novo SCLCs displayed a higher frequency of copy number loss or UPD than mutation. While copy number loss and UPD occurred frequently in both transformed SCLCs and de novo SCLCs, they were rare in primary LUADs. Moreover, we found that many high‐frequency mutated genes (such as *CLTCL1, LRIG3, PABPC1, PTCH1*) shared by transformed SCLCs and de novo SCLCs but excluded by primary LUADs, with their main type of mutation being either UPD or genomic losses as a “second hit” event. The high frequency UPD in transformed SCLC further supports the theory that transformed SCLC originates from lineage transformation. Moreover, the similarity of gene profiles between transformed SCLC and de novo SCLC might contain key factors in lineage‐specific drivers of lung neuroendocrine cell carcinoma.

Segmental UPD is commonly identified in tumors with impaired chromosome repair mechanisms. To be more detailed, we visualized the distribution of various kinds of CNV happened in genes belonging to six DNA damage repair (DDR) pathways (Figure S3A), including base excision repair (BER), nucleotide excision repair (NER), Fanconi anemia (FA), homology‐dependent recombination repair (HRR), non‐homologous end joining (NHEJ) and mismatch repair (MMR). As expected, UPD extensively happened in genes involved in DDR pathways, implying defective function of DNA repair in transformed SCLCs. In addition, considering the important role of the Notch signaling pathway in the determination of intra‐tumoral heterogeneity of SCLCs, we also visualized the distribution of different categories of CNV happened in genes of the pathway. UPD of genes involved in Notch signaling pathway was also predominantly found in transformed SCLCs, implying the relationship between loss of heterozygosity of these genes and unique features of transformed SCLCs (Figure S3B). Lastly, we focused on genes of the stem cell relevant pathway, as some antibody drug conjugates (ADCs) have been developed to target these genes for the treatment of SCLCs. Consistent with two pathways mentioned above, UPD of genes involved in this pathway was also predominantly found in transformed SCLCs (Figure S3C), which might provide more opportunities for drug development.

### 3.5 Potential Genomic Biomarkers Differentiating Transformed SCLCs From De Novo SCLCs

**FIGURE 4:**
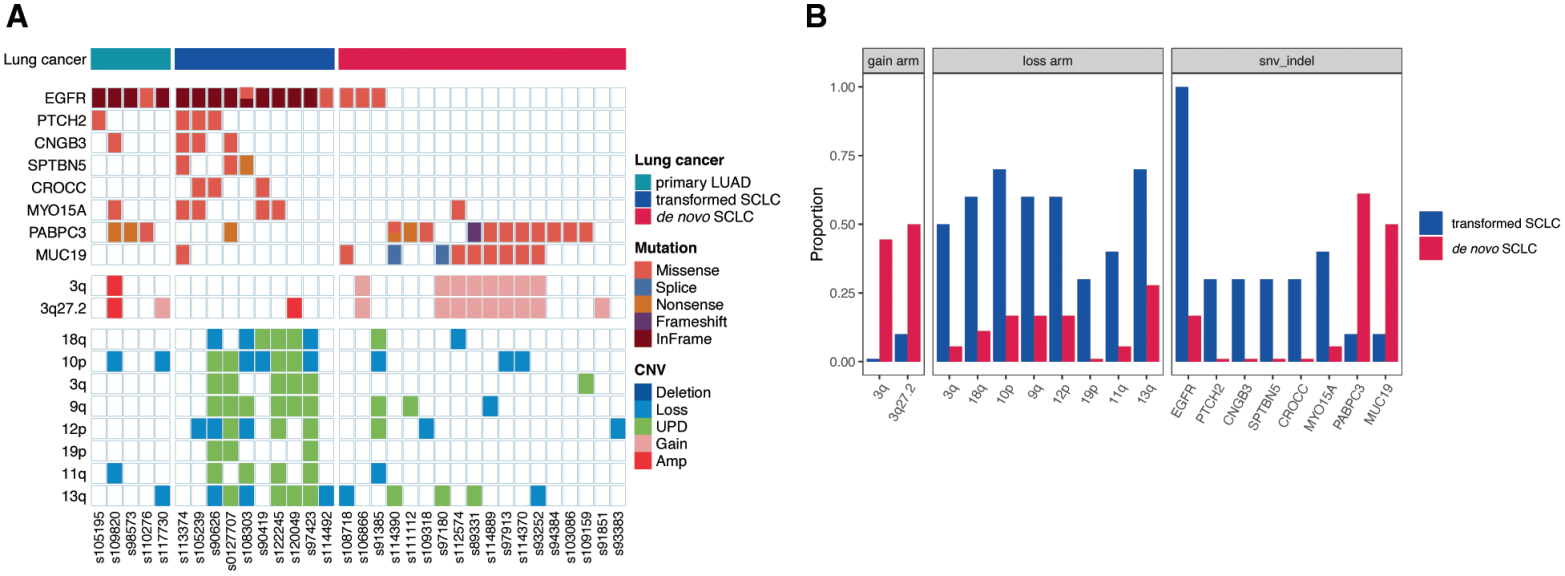
Potential biomarkers indicating transformed and de novo SCLCs. (A) Heat map showing the distribution of dominant mutated genes and somatic copy number alteration events in either transformed or de novo SCLCs. Rows representing genes and arm regions and columns representing samples. Types of mutation and somatic copy number alteration were shown in the legend. The color track on the top of heat map showing the pathology subtype. (B) Bar plot showing the proportion of patients with gained arm region, lost arm region and mutated gene in transformed and de novo SCLCs. Blue bar indicating transformed SCLCs, while red bar indicating de novo ones.

Unlike the high frequency of UPD events observed in both transformed SCLC and de novo SCLC, based on the G‐score distribution plot produced by GISTIC2, we found the pattern of peaks indicating deeply amplified and deleted genomic regions was quite different between transformed SCLCs and de novo SCLCs, without any overlap (Figure S4). First, at the level of mutated genes, we found that mutations in *EGFR*, *PTCH2*, *CNGB3*, *SPTBN5*, *CROCC*, and *MYO15A* were prevalent in transformed SCLCs, whereas mutations in *PABPC3* and *MUC19* were dominant in de novo ones (Figure 4A,B). Second, at the level of somatic copy number alterations, it was evident that genomic loss, especially for UPD events, in arm regions 18q, 10p, 3q, 9q, 12p, 19p, 11q, and 13q occurred at a striking higher frequency in transformed SCLCs compared to de novo ones, whereas genomic gain in the arm region 3q was predominantly observed in de novo SCLCs (Figure 4A,B). The clinical utility of these potential biomarkers should be confirmed in prospective clinical trials.

## 4 Discussion

Histological transformation from one subtype to another has been documented rare in lung cancer patients. In the largest cohort study involving 2624 rebiopsy cases, the incidence of transformation to SCLC was found to be 2.2%, compared to 0.6% for other NSCLC subtypes [13, 14, 15]. Although transformation from LUAD to SCLC is not the only variant observed, it is most frequently seen following EGFR‐TKI therapy [16]. Similar transformations have also been noted in patients with ALK and ROS1 mutations [17, 18]. However, the origin of histological transformation to SCLC has always been a subject of debate. Yet there is no consensus on the treatment of transformed SCLC.

In this study, Whole‐exome sequencing was performed on tissues of primary LUAD with EGFR mutation before SCLC transformation occur, transformed SCLC tissue after EGFR‐TKI treatment, and de novo SCLC to elucidate their similarities, differences, and connections. As result, our evidence is more supportive of the conversion hypothesis and possible potent treatment suggestion for transformed SCLC has also been speculated.

In our cohort, all cases of the transformed SCLC inherited the EGFR mutation from the pre‐transformed LUAD (Figure 1) and mPFS before SCLC transformation is 17.8 months from either 1st or 3rd generation EGFR‐TKIs. Our results also showed transformed SCLCs exhibited a large number of CNV losses as in de novo SCLCs and contained partial CNV gains as seen in primary LUADs (Figure 3A,B). Meanwhile, transformed SCLCs exhibited a large number of UPDs (Figure 2, Figure 3C). These indicate that SCLC may evolved from a lineage evolution rather than selection through EGFR‐TKI treatment. In case of mixed LUAD and SCLC, they have different origins with different genomic characteristics and biological behavior, according to the theory of dominance screening. If SCLC was transformed from LUAD, the genomic characteristics of transformed SCLCs tended to be more similar with primary LUADs than de novo SCLCs. However, some scholars cited studies in which primary mixed adenocarcinoma and small cell were detected, cells of two tissue types had the same mutation, suggesting that they were derived from common progenitor cells [19]. It also suggests that cancer cells have plasticity under pressure selection. It is widely believed that NSCLC mainly occurs from gain‐of‐function mutations by oncogenes, whereas SCLC mainly occurs from loss of function of tumor suppressor genes from the existing large number of sequencing studies on NSCLC and SCLC [20]. In our result, transformed SCLCs exhibited a large number of CNV losses, which also occur in de novo SCLCs. At the same time, transformed SCLCs contained partial CNV gains as seen in primary LUADs (Figure 3A,B). Meanwhile, transformed SCLCs exhibited a large number of UPDs (Figure 2, Figure 3C). According to the occurrence characteristics of SNPs in UPD, it seems that transformed SCLCs have suffered a second genomic mutation, consistent with the two‐hit hypothesis. This also suggests that SCLC is of a transformed origin.

On the other hands, 2 out of 7 patients had a TP53 mutation prior to transformation, representing 28.6%. In contrast, it is significantly higher in transformed SCLC tissues at 80% (8 out of 10). It indicates that the majority of TP53 mutations were newly acquired during transformation. Both TP53 and RB1 deletions are critical for the emergence of de novo SCLC [7, 16], and our result support their loss also plays a significant role in the tumorigenesis of transformed SCLC. In particular, it has been shown in multiple case reports that therapy‐induced RB1 loss in tumor tissues is a necessary condition for tissue transformation [21, 22]. However, the presence of wild type of TP53 and RB1 in the pre‐transformed LUAD stage does not predict that histological transformation will not occur in the future. In addition to RB1 loss, c‐Myc activation is also considered a key factor in the lineage transformation of lung cancer [23]. For the first time, Scientists had constructed histological transformation model from AT2 cells and neuroendocrine cells to SCLC in the animal, mimicking the naturally occurring small cell transformation process in patients [10]. AT2 cells are considered the origin of LUAD cells and can undergo histological transformation to SCLC under the combination of TP53/RB1 inactivation, Myc activation, and EGFR downregulation. In our cohort, as shown in the G‐score distribution plot, transformed SCLCs exhibited a deeply amplified genomic region at 8q24, where c‐Myc is located (Figure S4).

Identifying the stage of the transformed SCLC independent from LUAD is crucial for predicting patient survival and informing clinical treatment decisions. Within our cohort, there were four patients with limited‐stage and eight with extensive‐stage. Among the four patients, three are still alive as of the last follow‐up, with an OS of 21.0 months. Among the eight patients, only one patient is alive, with a median OS (mOS) of 5.0 months. To the best of our knowledge, this is the first study emphases the necessity to distinguish limited‐stage and extensive‐stage in transformed SCLC in order to predict survival and implement different treatment strategies. However, in order to perform precise staging, it is necessary to perform sufficient biopsy on the progressing lesions to determine whether they are small cell component or adenocarcinoma component progression.

When it comes to treatment strategies for transformed SCLC, current options are controversial [24]. The genomic mutation landscape reflects that the gene mutation characteristics of transformed SCLC are intermediate between primary LUADs and de novo SCLCs, leading to uncertainty over whether the treatment plan should follow LUAD or SCLC, as current clinical guidelines have not given clear recommendations. In our previous study, overall response rate (ORR) of etoposide‐containing chemotherapy was 38.5% (5/13) [25]. Another retrospective study showed ORR to platinum‐etoposide was 54% (*n* = 46) and to taxane‐containing regimens was 50% (*n* = 20) in transformed SCLC and other neuroendocrine carcinomas [24]. These indications suggest that the efficacy to chemotherapy in this type of cancer also exhibits behavior of both small cell and adenocarcinoma. However, the study also observed none of 17 patients respond to a checkpoint inhibitor, either a single‐agent programmed death‐1 or programmed death‐ligand 1 inhibitor (*n* = 9) or as part of the combination ipilimumab‐nivolumab regimen (*n* = 8). In our result, the TMB was significantly lower in transformed SCLCs compared to de novo ones (Wilcoxon rank sum test, *p* value = 0.01). High TMB is closely related to the better efficacy of immunotherapy, suggesting that transformed SCLC may be insensitive to immunotherapy, which is consistent with the result in the above study. However, it doesn't mean combined treatment with immunotherapy cannot bring benefit to these patients. Yang Jinji et al. showed that in the I/O group, ORR was 73% (8/11) which is higher than former studies, among them nine patients received atezolizumab‐bevacizumab‐carboplatin‐paclitaxel (albumin‐bound) (ABCP), and 2 received atezolizumab‐etoposide‐carboplatin (ECT) [26]. So that, Huang Jie et al. classified transformed SCLC into two subtypes upon RB1 gene status. The RB1 wild‐type subgroup may be more sensitive to immunotherapy, while the RB1 mutant subgroup may be suitable for PI3K/AKT pathway inhibitors [27].

As a matter of fact, de novo SCLC has some new therapeutic agents targeting its characteristic gene targets, such as targeted therapies based on the DDR signaling pathway and Notch signaling pathway. In our results, we found a high degree of similarity between transformed SCLCs and de novo SCLCs in gene alteration from these two pathways (Figure S4), suggesting that these emerging treatment modalities may also be applicable to transformed SCLCs. A promising approach involves the application of olaparib, a poly ADP ribose polymerase (PARP) inhibitor, which specifically targets mutations in DDR signaling. At present, a Phase II trial is underway assessing the combination of a PD‐L1 inhibitor and olaparib in patients with EGFR mutation‐positive transformed SCLCs (NCT04538378), with positive clinical outcomes expected.

## 5 Conclusions

In conclusion, the genomic characterization of transformed SCLC presented in our study contributes to a deeper understanding of this disease subtype and may pave the way for more effective diagnostic and therapeutic approaches in the management of EGFR‐mutated lung cancers that undergo transformation, potentially improving patient outcomes in this challenging disease context.

## Author Contributions

**Jinjing Tan:** conceptualization (equal), funding acquisition (equal), writing – original draft (lead). **Dan Zhao:** formal analysis (lead), methodology (lead). **Qunhui Wang:** software (equal). **Yanjing Peng:** software (equal). **Jie Li:** validation (equal). **Xi Li:** validation (equal). **Nanying Che:** project administration (equal), resources (lead). **Ying Hu:** project administration (equal), writing – review and editing (equal). **Hua Zheng:** conceptualization (equal), funding acquisition (equal), writing – review and editing (equal).

## Ethics Statement

This study received approval from the Ethics Committee of Beijing Chest Hospital, affiliated with Capital Medical University (approval number: 2021‐43‐2).

## Consent

Informed written consent was obtained from all participants prior to any study‐related procedures.

## Conflicts of Interest

The authors declare no conflicts of interest.

## Supporting information


Data S1.

## Data Availability

All relevant data generated during this study will be made available by the corresponding authors upon reasonable request.
