# Supplementary material for: Whole Exome Sequencing Study Identifies Distinct Characteristics of Transformed Small Cell Lung Cancer With EGFR Mutation Compared to De Novo Small Cell and Primary Non‐Small Cell Lung Cancers
Source: Cancer Med. 2025 Apr 8;14(7):e70838. doi: 10.1002/cam4.70838 (PMC11976457; doi:10.1002/cam4.70838)
Supplement: Supplementary file 1 — Data S1. [file CAM4-14-e70838-s001.docx]

**
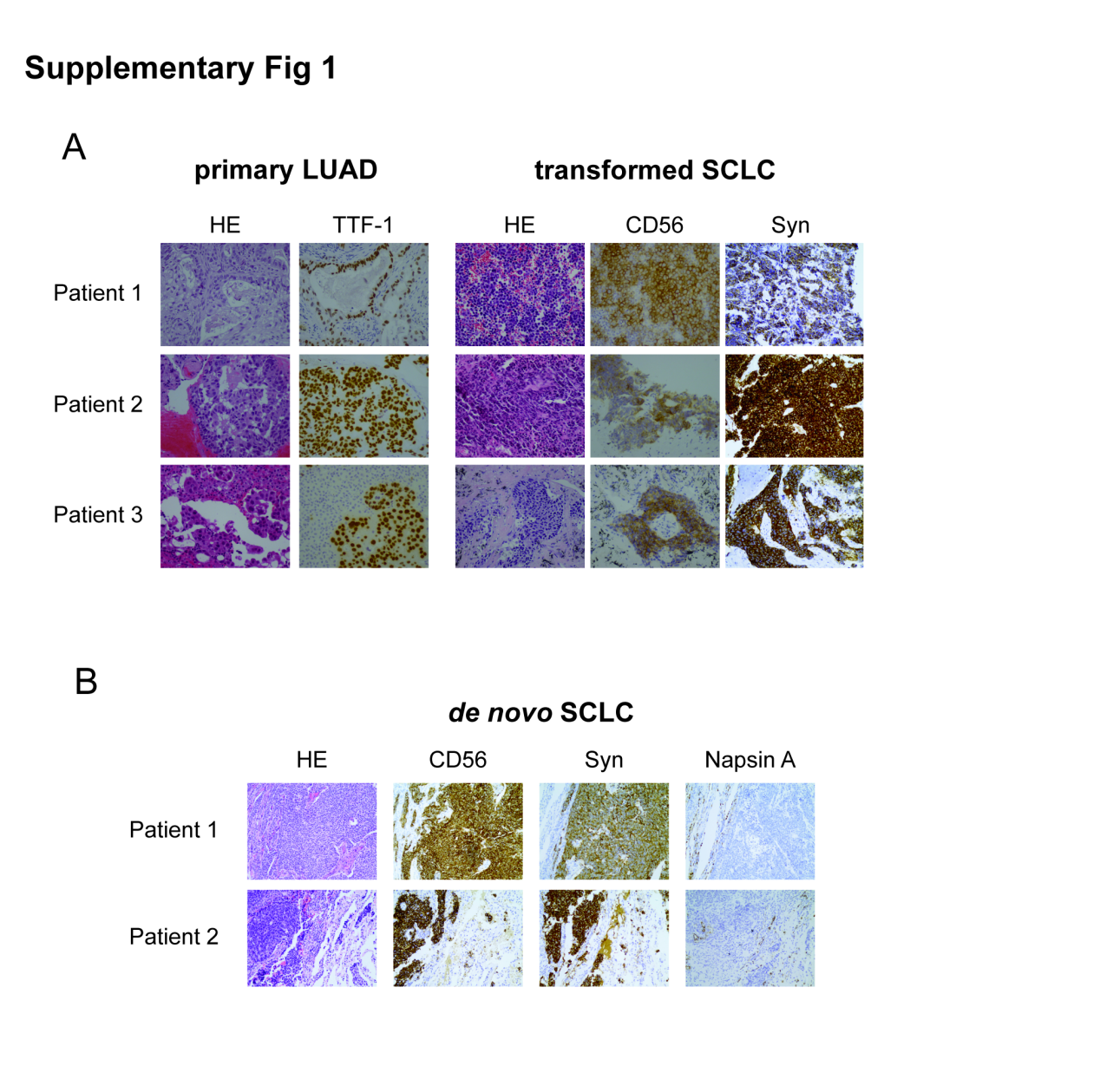
**

**Supplementary Fig. 1** Histological morphology of primary LUADs before transformed, transformed SCLCs and *de novo* SCLCs. **A** Three patients with small cell transformation were randomly selected. Their pre-transformated primary LUAD and post-transformated SCLC Formalin-Fixed Paraffin-Embedded (FFPE) tissue were performed with H&E staining (left) and immunohistochemical staining (right). **B** Two *de novo* SCLC PPFE tissue were performed with H&E staining and immunohistochemical staining for SCLC markers. Immunohistochemical staining used the lung adenocarcinoma biomarker TTF-1 and the SCLC biomarker CD56, Syn and Napsin A. H&E, hematoxylin and eosin; Syn, Synaptophysin.

**
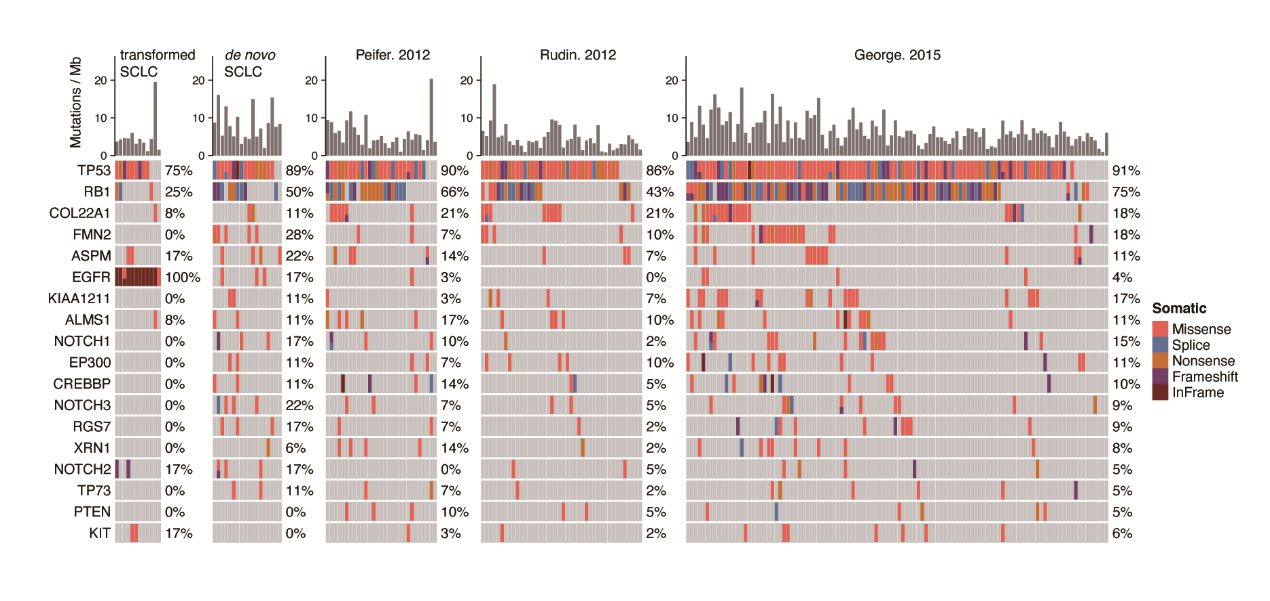
**

**Supplementary Fig. 2** Somatic mutation landscape of published *de novo* SCLCs. Five heatmaps showing somatic mutation landscape of transformed and *de novo* SCLCs in our research, *de novo* SCLCs in Peifer et al. 2012, Rudin et al. 2012 and George et al 2015. Rows representing genes and columns representing samples from different cohorts. The prevalence of mutated genes was labeled in the right of each heatmap, while TMB of patients was displayed on the top of each heatmap. Somatic mutation category was shown in the legend. TMB, tumor mutational burden.


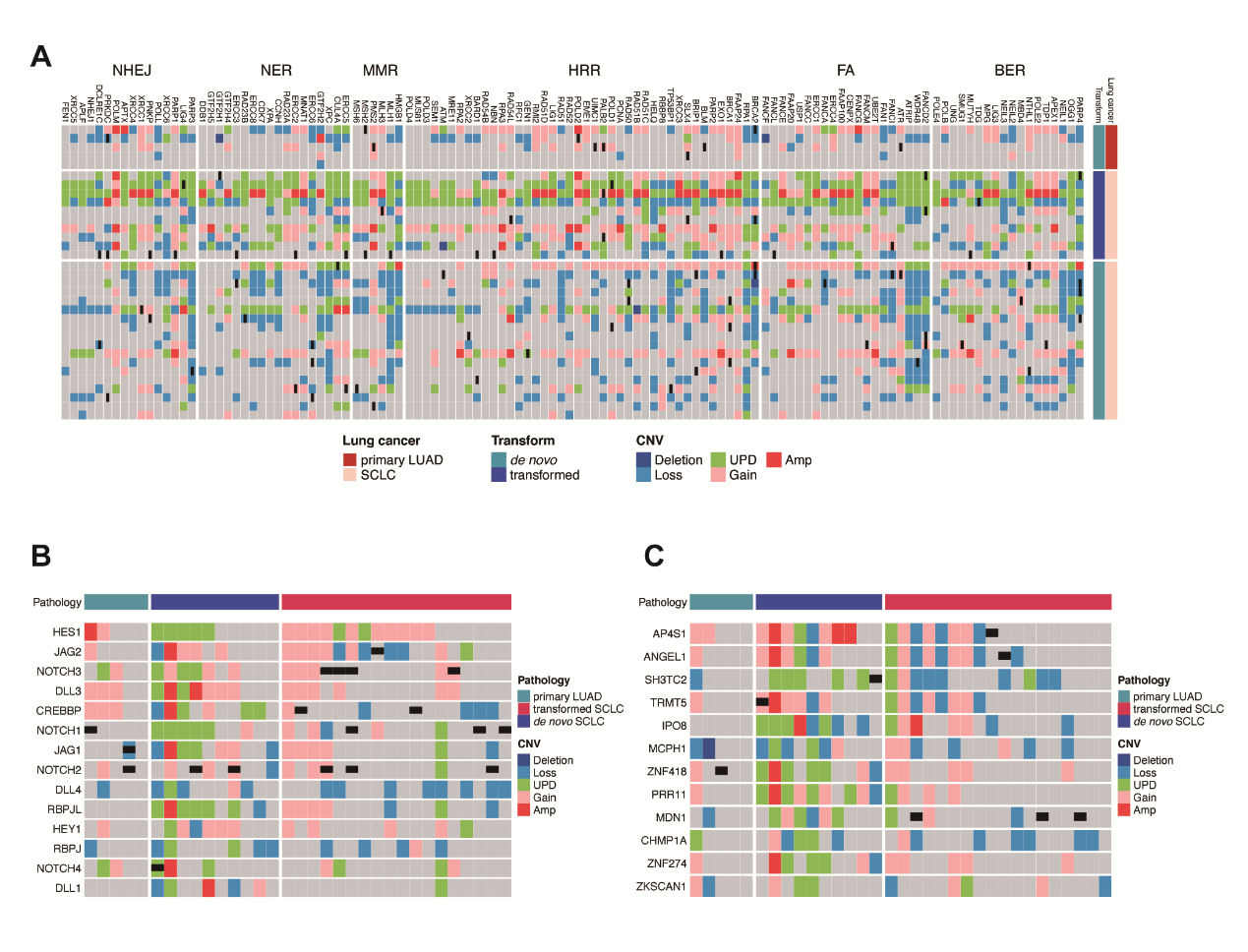


**Supplementary Fig. 3** Somatic copy number alteration landscape of genes involved in different pathways. **A-C** Three heatmaps showing somatic copy number alteration landscape related to DDR, Notch signaling and stem cell relevant pathways, respectively. Rows representing genes with pathway annotation and columns representing samples. On the top of heatmap, color track showing the cohort of origin for samples. Categories of somatic copy number alteration was shown in the legend. DDR, DNA damage response.


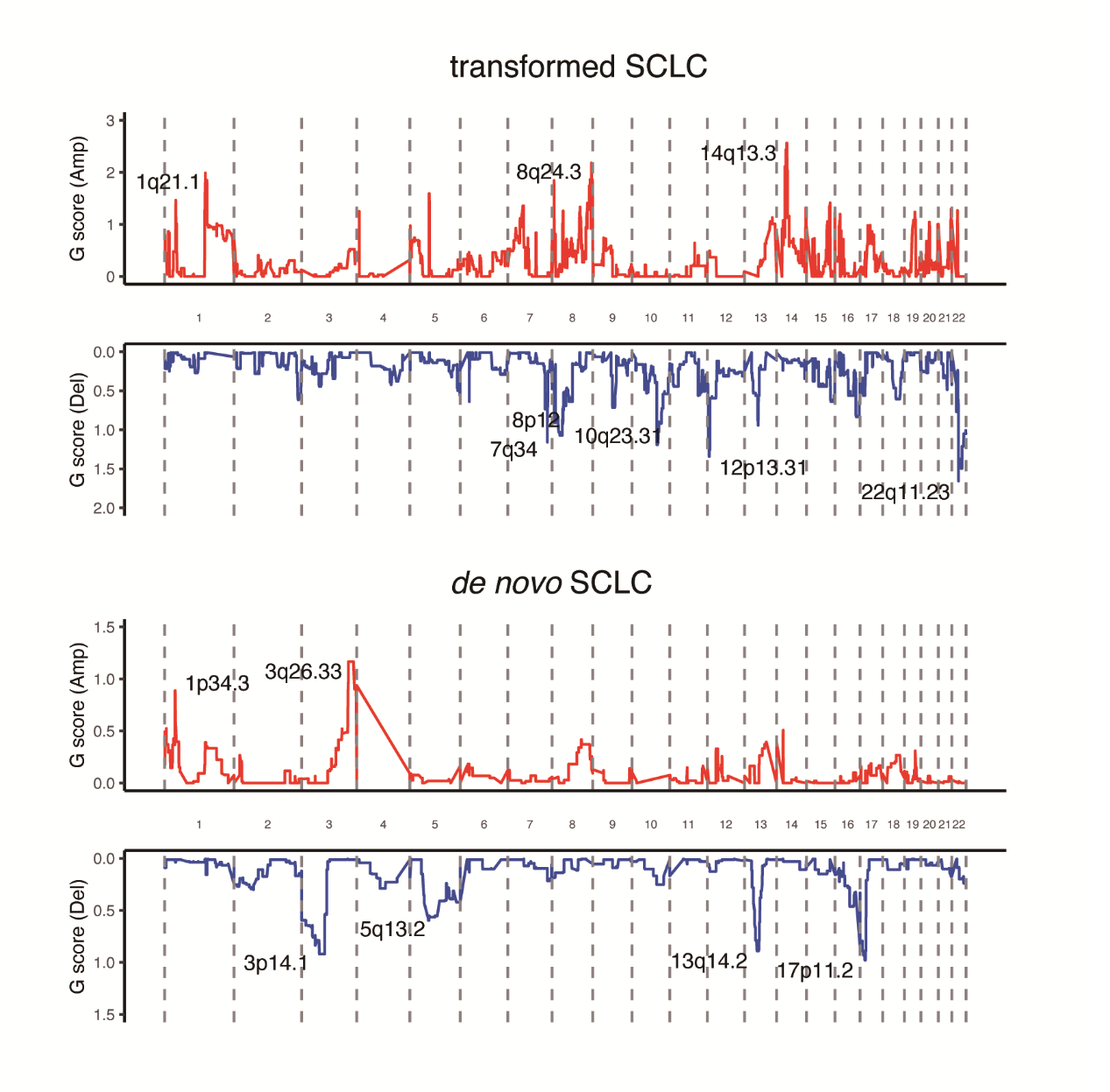


**Supplementary Fig. 4** Genome-wide distribution of CNV peaks in *de novo* and transformed SCLCs. Line plot showing the distribution of G-score for genomic amplification and deletion in two cohorts. G-score indicates the sum of amplification or deletion amplitudes through the cohort. Arm regions of chromosomes with significantly high G-score determined by permutation testing were labeled in the plot. CNV, copy number variation.

**Supplementary Table 1** Clinical and pathological parameters of primary LUAD, transformed SCLC and *de novo* SCLC patients

| Parameters | Category | primary LUAD | Transformed SCLC | *de novo* SCLC |
| --- | --- | --- | --- | --- |
| Total |  | 5 | 12 | 18 |
| Gender | Male | 1 | 2 | 15 |
|  | Female | 4 | 10 | 3 |
| Age | > 60 | 3 | 7 | 8 |
|  | ≤ 60 | 2 | 5 | 10 |
| Smoking | yes | 1 | 2 | 13 |
|  | no | 4 | 10 | 5 |
| TNM Stage | I | 0 | 1 | 4 |
|  | II | 1 | 0 | 8 |
|  | III | 0 | 2 | 6 |
|  | IV | 4 | 9 | 0 |
| SCLC stage | Limited |  | 4 | 18 |
|  | Extensive |  | 8 | 0 |
| EGFR mutation | Exon 19 del | 11 | 11 | 0 |
|  | L858R | 1 | 1 | 2 |
|  | others | 0 | 0 | 1 |
|  | none | 0 | 0 | 15 |

LUAD lung adenocarcinoma; SCLC small-cell lung cancer; TNM tumor node metastasis; EGFR epidermal growth factor receptor

**Supplementary Table 2** Clinical information of transformed SCLC patients

| Patient ID | Gender | Age | EGFR mutation type | primary LUAD stage | Treatment before transformation | Transformed SCLC stage | Transform time (Month) | Pathology after transformation | Purity% | WES QC |
| --- | --- | --- | --- | --- | --- | --- | --- | --- | --- | --- |
| Patient 01 | Female | 65 | 19del | IV | Chemotherapy, Getifinib | Extensive | 12 | Mixed LUAD and SCLC | 88 | pass |
| Patient 02 | Female | 62 | 19del | IV | Icotinib | Extensive | 12 | Pure SCLC | 40 | pass |
| Patient 03 | Female | 64 | 19del | IV | Erlotinib, chemotherapy, ASK120067 | Extensive | 17 | Pure SCLC | 100 | pass |
| Patient 04 | Female | 69 | 19del | I | Icotinib | Limited | 9 | Pure SCLC | 100 | pass |
| Patient 05 | Male | 72 | 19del | IV | Gefitinib | Extensive | 6 | Pure SCLC | 33 | not |
| Patient 06 | Female | 63 | 19del;T790M | IV | Chemotherapy, icotinib, osimertinib | Extensive | 65 | Mixed LUAD and SCLC | 100 | pass |
| Patient 07 | Female | 72 | L858R | IV | Icotinib | Extensive | 6 | Mixed LUAD and SCLC | 51 | pass |
| Patient 08 | Male | 54 | 19del | IIIB | Icotinib | Limited | 10 | Mixed LUAD and SCLC | 100 | pass |
| Patient 09 | Female | 60 | 19del;T790M | IV | Icotinib, osimertinib | Extensive | 41 | Pure SCLC | 30 | pass |
| Patient 10 | Female | 46 | 19del | IIIA | icotinib | Limited | 7 | Pure SCLC | 86 | not |
| Patient 11 | Female | 56 | 19del | IV | Icotinib, osimertinib | Extensive | 25 | Mixed LUAD and SCLC | 58 | pass |
| Patient 12 | Female | 42 | 19del | IV | osimertinib | Limited | 12 | Mixed LUAD and SCLC | 80 | pass |

SCLC, small-cell lung cancer; EGFR, epidermal growth factor receptor; LUAD, lung adenocarcinoma; WES QC, whole-exome sequencing quality control.

**Supplementary Table 3** EGFR and TP53 mutation of paired primary LUAD and transformed SCLC

| Patient ID | Gene mutation of primary LUAD | | Gene mutation of transformed SCLC | |
| --- | --- | --- | --- | --- |
|  | EGFR | TP53 | EGFR | TP53 |
| Patient 01 | 19del | Wild type | 19del | R273C |
| Patient 02 | 19del | Wild type | 19del | S183T |
| Patient 03 | 19del | Wild type | 19del | Wild type |
| Patient 04 | 19del | Wild type | 19del | R158H |
| Patient 05 | 19del | Unknown | 19del | Unknown |
| Patient 06 | 19del; T790M | Unknown | 19del; T790M | C229fs |
| Patient 07 | L858R | Unknown | L858R | Wild type |
| Patient 08 | 19del | S215N | 19del | S215N |
| Patient 09 | 19del; T790M | Q136E | 19del | Q136E |
| Patient 10 | 19del | Wild type | 19del | Unknown |
| Patient 11 | 19del | Unknown | 19del | V143Rfs* |
| Patient 12 | 19del | Unknown | 19del | M246T |

EGFR epidermal growth factor receptor; TP53 tumor protein 53; LUAD lung adenocarcinoma; SCLC small-cell lung cancer
